# A modelling analysis of a new multi-stage pathway for classifying achievement of public health milestones for leprosy

**DOI:** 10.1098/rstb.2022.0408

**Published:** 2023-10-09

**Authors:** Emma L. Davis, Ron E. Crump, Graham F. Medley, Anthony W. Solomon, Venkata Ranganadha Rao Pemmaraju, T. Déirdre Hollingsworth

**Affiliations:** ^1^ Mathematics Institute, University of Warwick, Coventry, CV4 7AL, UK; ^2^ London School of Hygiene & Tropical Medicine, London, WC1E 7HT, UK; ^3^ Global Neglected Tropical Diseases Programme, World Health Organization, Geneva, 1211, Switzerland; ^4^ Global Leprosy Programme, World Health Organization, New Delhi, India; ^5^ Big Data Institute, Li Ka Shing Centre for Health Information and Discovery, University of Oxford, Oxford, OX3 7LF, UK

**Keywords:** leprosy, Hansen's disease, elimination, interruption of transmission, milestones, modelling

## Abstract

Several countries have come close to eliminating leprosy, but leprosy cases continue to be detected at low levels. Due to the long, highly variable delay from infection to detection, the relationship between observed cases and transmission is uncertain. The World Health Organization's new technical guidance provides a path for countries to reach elimination. We use a simple probabilistic model to simulate the stochastic dynamics of detected cases as transmission declines, and evaluate progress through the new public health milestones. In simulations where transmission is halted, 5 years of zero incidence in autochthonous children, combined with 3 years of zero incidence in all ages is a flawed indicator that transmission has halted (54% correctly classified). A further 10 years of only occasional sporadic cases is associated with a high probability of having interrupted transmission (99%). If, however, transmission continues at extremely low levels, it is possible that cases could be misidentified as historic cases from the tail of the incubation period distribution, although misleadingly achieving all three milestones is unlikely (less than 1% probability across a 15-year period of ongoing low-level transmission). These results demonstrate the feasibility and challenges of a phased progression of milestones towards interruption of transmission, allowing assessment of programme status.

This article is part of the theme issue ‘Challenges and opportunities in the fight against neglected tropical diseases: a decade from the London Declaration on NTDs’.

## 1. Introduction

Leprosy, otherwise known as Hansen's disease, is a leprosy (NTD) caused by the bacteria *Mycobacterium leprae* or *M. lepromatosis*. Although it is curable if caught in the early stages, untreated disease can lead to permanent disability, affecting the skin and peripheral nerves [1]. The majority of new cases are detected in southeast Asia, but cases still occur across all six World Health Organization (WHO) regions. For many countries, most cases are imported, but there are a number of countries where incidence has declined from historically high levels to less than 10 cases per year in some districts for greater than 10 years and are likely to be on the pathway to elimination [2]. Transmission is mainly controlled using case detection and treatment with multi-drug therapy (MDT), although tracing of contacts and their pre-emptive treatment is also recommended [1] to reduce the impact of uncertain infection status, long incubation periods and detection delays [3]. While new tools for diagnosis, prevention and control have been in development, the mainstay of control remains investigating and treating detected cases [4].

Although global elimination of leprosy as a public health problem (defined as a registered prevalence of less than 1 per 10 000 population globally) was achieved in 2000, and this threshold was achieved in most countries by 2010, more than 100 000 new cases are still reported annually worldwide. In 2021, the WHO published a strategy targeting, by 2030, a 70% reduction in the annual number of new cases detected and 120 countries detecting zero new autochthonous cases [5,6].

There is an evident gap between low incidence and non-endemic status (defined as when leprosy is not normally present among the autochthonous population in the area or country, but sporadic cases may occur) [7], although progress is being made towards that more advanced target: in 2019, 45 countries detected zero new cases and 99 countries detected fewer than 1000 new cases [1]. However, setting verifiable criteria for classifying non-endemic status is a problem that has proven challenging for a number of diseases due to the random fluctuations that tend to occur at low incidence levels [8,9]. Measuring progress towards leprosy elimination is further obstructed by the long incubation periods and detection delays characteristic of the disease, meaning that new cases could represent transmission events from 10 or more years prior to detection. Such delays may also have been exacerbated due to reductions in case detection and control activities across all NTDs in 2020–2022, due to the COVID-19 pandemic [10,11].

Due to the delay from symptom onset to diagnosis, and the difficulties around identifying duration and timing of exposure, measurement of leprosy's incubation period can be challenging, which in turn affects the interpretation of incidence of diagnosis. A previous modelling study used data from cases diagnosed in military service personnel living in non-endemic communities who had had short exposure periods associated with limited periods of time in endemic countries [12], finding a modal incubation period of 3.8 years, but with some incubation periods lasting more than 20 years. Other studies demonstrate a mean detection time of 1–8 years post-symptom onset, with fear of stigma and a lack of pain accompanying symptoms being strong predictors of longer detection delays [13,14].

Despite the challenges of interpreting highly stochastic low-incidence dynamics, it is important to provide a framework with which to interpret progress towards the elimination target, to maintain political momentum [15]. The history of malaria control and elimination has shown how the methods of measurement have changed over time [16]. Mathematical modelling can provide support in this area, such as developing tools to interpret low case numbers. Approaches include methods for differentiating small outbreaks of malaria from imported cases [17] and for classifying repeat findings of zero infections among surveys for sleeping sickness [18]. Other methods such as critical slowing down theory focus on understanding the peculiar dynamics of the tail end of any transmission process, where, inevitably, the final cases are those with the longest incubation periods [19,20].

In July 2023, the WHO published new technical guidance on interruption of transmission and elimination of leprosy disease [21], which is accompanied by a leprosy elimination monitoring tool [22] that lays out a phased approach to monitoring progress towards interruption of transmission, elimination of leprosy disease and non-endemic status with the aim of promoting a ‘bottom-up’ method for building the evidence that non-endemic status is achieved. Within the monitoring tool there are a number of examples of sub-national areas where new case detection or incidence has been low for many years, and shows how the tool assists in evaluating progress.

Modelling work has projected a continuation of the progressive downward trend in incidence observed in most countries [23,24], while also demonstrating that there is likely to be a substantial pool of undiagnosed infection and highlighting the need for active case detection and contact tracing [25]. However, the key metric used in targets and modelling studies is the new case detection rate (NCDR), which is an increasingly poor indicator of trends in transmission as we get closer to true transmission interruption [25]. This enhances the importance of developing new tools and metrics for classifying the final stages of elimination.

In this study, we use a probabilistic model based on previously fitted incubation period and detection delay distributions to investigate the sensitivity and specificity of milestones for classifying non-endemic status that could be implemented at evaluation unit (EU) level. The WHO monitoring and evaluation tool states that an EU may be differently defined in each setting depending on the dynamics of leprosy and availability of data. For example, EUs could be provinces, districts or even villages. Our aim is to evaluate under what conditions these guidelines may or may not identify halting of transmission given our limited knowledge of the epidemiology of this disease. In order to do this, we simulate scenarios representing both a decline in transmission incidence to zero new transmissions and low-level persistent transmission, and evaluate the sensitivity and specificity of this approach.

### (a) Leprosy elimination framework

**Figure 1.**
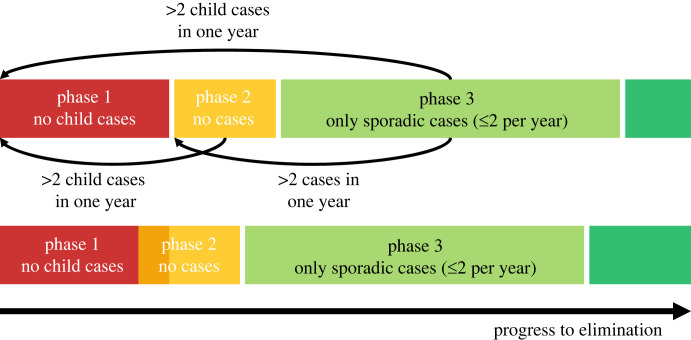
Elimination phases. Schematic of the WHO leprosy elimination framework. Phase 1: 5 years with no new autochthonous cases in children. Phase 2: 3 years with no new autochthonous cases (can overlap with phase 1 as shown in lower schematic). Phase 3: 10 years with only sporadic autochthonous cases (must start after phase 2). Reversal to phase 1 if greater than 2 sporadic child cases in 1 year. Reversal to phase 2 if greater than 2 sporadic cases (any) in one year. Dark green: non-endemic status, achieved after passing out of all phases.

The phases process presented in the aforementioned technical guidance published in July 2023 is outlined here (figure 1) [21,22]. For the appropriate spatial scale, incidence can pass through the following phases, with the possibility of going backwards as well as forwards through the phases. The technical guidance provides extensive context regarding the complexities of gathering rigorous, quality-assured data in the circumstances of an elimination programme. There is also important discussion in the guidance of the provision of services and the role of both passive and active screening in the different phases. For our analysis, we focus on the dynamics of the resulting detected cases.

*Phase 1—until interruption of transmission*. This phase is expected to have a long time span, but there may be areas where child cases have not occurred for many years. The milestone to move to the next phase is ‘no new autochthonous cases among children for at least 5 consecutive years’.

*Phase 2—interruption of transmission until elimination of disease.* During the next phase, only autochthonous cases are detected. The WHO technical guidance notes that there are sporadic cases in children in some areas which have passed into this phase, but these do not appear to have led to re-emergence of leprosy. It also notes that there may be clustering of cases within families or close contacts on the pathway to elimination. The milestone to move to the next phase is ‘no new autochthonous cases for at least three consecutive years (and no child cases in 5 years)’.

*Phase 3—post-elimination surveillance.* Following a verification of elimination of transmission by WHO, phase 3 begins, in which very low incidence may still be detected. The milestone for moving to the next phase is ‘no or only sporadic autochthonous cases for a period greater than or equal to 10 years’.

*Non-endemic status*, when leprosy is not normally present in the area or country, is the final status. Sporadic cases may occur due to the long incubation period of leprosy.

In our analysis, we consider hypothetical scenarios for underlying declines incidence of infection and model how they would result in detected cases using previously published distributions for the incubation period and time from symptom onset to detection, to consider how these scenarios would lead to progression through the phases described above. We evaluate (i) the sensitivity of this approach as a simulation achieves the milestones over the years following the halting of transmission, and (ii) the specificity of this approach in the years following a decline but not complete cessation of transmission. We also investigate the second output for different levels of ongoing transmission and both outputs for different time periods after the first milestone is achieved.

## 2. Methods

To investigate the potential sensitivity and specificity of different criteria for elimination milestones, we use a simple probabilistic model to consider the dynamics of observed/detected incidence of infection over the decades following two separate population-level transmission scenarios: (i) halted transmission (disease no longer endemic) and (ii) low-level persistence of transmission. As discussed above, the technical guidance outlines that the particular spatial scale at which the evaluation takes place depends on the local dynamics of leprosy and the scale of availability of data. Therefore, we characterize the population at risk as being in a particular EU.

**Figure 2.**
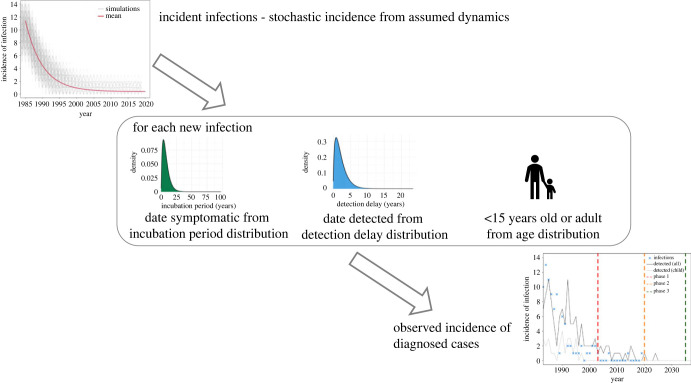
Schematic of the model—incident infections are generated under a particular scenario. Each infection is then allocated a time to symptoms and detection, as well as an age (see Methods), resulting in emerging dynamics of incidence of diagnoses.

We first simulate the incidence of infection in each of these scenarios, and then for each of these infections we use published distributions for the incubation period and the time from symptoms to detection, or detection delay, to simulate the annual incidence of the diagnosis of cases (figure 2). In brief, we randomly allocated the ages of new infections according to an age distribution; for each case, we then added age at infection to the incubation period and detection delay, which were assumed to be independent, leading to classification of each case as either a child (aged less than 15 years) or an adult case at the time of diagnosis, using WHO classification standards [5]. For longer incubation periods and longer detection delays, cases are less likely to be detected as children.

More precisely, we first simulated incident infections in a particular EU. For the halted transmission scenario, we simulated the decline in infections prior to a halt in local transmission, represented by no new incident infections, within an EU as an exponential decline in transmission incidence from a mean of 10 infections per year (range: 0–20, s.d.: 2.24) to zero infections per year across a 35-year period, at an annual rate of decline of 0.2. When expected incidence has been under 0.01 for 5 years, the exponential function is replaced with zero. The second scenario, low-level persistent transmission despite a successful decline in transmission, is simulated as an exponential decline in transmission incidence from 10 infections per year to a low level across a 35-year period, representing the same annual rate of decline as the first scenario. We investigated the effect of the value of this low-level transmission, such as a range of mean annual incidences between 0.2 and 4 infections per year (20 scenarios in total), with 2 infections per year (range: 0–10, s.d.: 1.34) representing a typical low-level persistence scenario.

**Figure 3.**
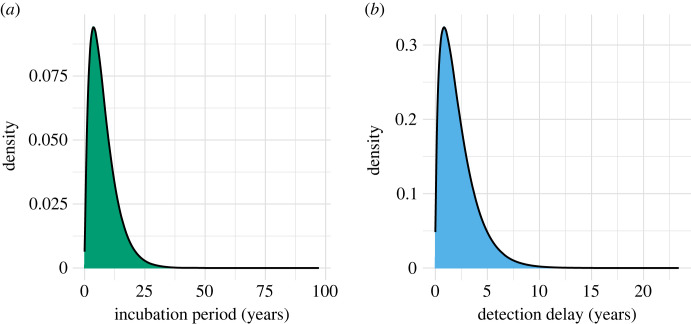
Delay distributions. Fitted gamma distributions for (*a*) the incubation period, from infection to symptoms and (*b*) detection delay, from symptoms to detection. Incubation period: shape = 1.92, rate = 0.247. Detection delay: shape = 1.60, rate = 0.714. All values to 3 significant figures.

We used R Statistical Software v4.2.2 [26] to run 5000 simulations for each distinct scenario, using binomial sampler rbinom from the core stats package. Since incidence is low, this will be similar to incidence using a Poisson distribution, while giving a constraint on the upper bound of the number of cases observed, informed by the examples in the leprosy elimination monitoring tool [22]. Once we had a simulated pattern of infections, we then simulated incubation periods and detection delays sampled from gamma distributions previously fitted by Crump and Medley [12] to generate the annual incidence of new leprosy diagnoses, shown in figure 3. The incubation period distribution (shape = 1.92; mean = 7.77) was fitted to data derived from veterans who contracted leprosy upon returning to the USA after serving in endemic areas [27,28]. The detection delay distribution (shape = 1.60; mean = 2.24) was based on patient cohort data from Bangladesh [29], which is likely to be more representative of the detection rate in endemic countries and is therefore used instead of the distribution previously fitted to the data on USA veterans. We assume that both these distributions remain constant through the period of simulation, which may be decades, and consider sensitivity to these assumptions. However, it is of course possible that there may be large changes in detection delays over the course of a long programme [12].

Due to the long mean delay from infection to detection (10.0 years, 95% CI: 2.19–24.6), we also considered ageing and death of infected individuals to classify cases as child (aged less than 15 years) or adult cases at the point of detection and to account for any right censoring due to death occurring prior to detection. We modelled the population using a population age-structure representative of sub-Saharan Africa [30]. It was beyond the scope of this work to consider a fully dynamic age distribution, which may be required for considering particular populations. Full simulation methods and associated R code are provided in the linked GitHub repository.

It is important to note that this is not a transmission model—we assume an underlying incidence and then simulate forward its consequences for detection of cases. This is due to the highly stochastic nature of incidence in a system in which incidence is low and there are such variable delay distributions, as illustrated by the examples in the leprosy elimination tool [22] where there are low numbers of cases over many years in some settings. These data are extremely challenging, or potentially impossible, to fit a transmission model to, and therefore we have gone for a scenario-based approach to evaluate the relationship between the scenario for very low levels of incidence and emergent detected cases.

For each simulation, we considered the three potential public health milestones that could be used to classify the stages of transmission reduction and elimination within an EU, labelled as passing out of phases 1–3 (figure 1). Passing out of phase 1 would require five consecutive years of detecting no autochthonous cases in children aged less than 15 years. Passing out of phase 2 would require three consecutive years of no autochthonous cases in adults or children; this period would be permitted to overlap with the 5-year period of no child cases to pass out of phase 1, meaning it would be possible to pass out of phase 1 and phase 2 in a total of 5 years. Following passing out of phase 2, a separate 10-year window of no, or only sporadic (less than or equal to 2), autochthonous cases would be required to pass out of phase 3. If at any point more than two autochthonous cases were detected in one year, this would mean a reversion to: before phase 2 if two or fewer of these cases were in children; or before phase 1 if more than two cases were in children.

Example scenarios, including demonstration of when any phase would be passed or reversed, are provided in electronic supplementary material, File 2.

For the purposes of analysing the usefulness of these proposed milestones for classifying non-endemic status, we define the *n*-year sensitivity as the proportion of halted transmission scenarios that pass a phase within *n* years of the final new infection. Similarly, we define the *n*-year specificity as the proportion of non-elimination scenarios that do not pass a phase across an *n*-year period of low-level persistence.

## 3. Results

### (a) Scenario 1. Halted transmission

For a scenario representing halted transmission, passing out of phase 1 by recording 5 years of no autochthonous cases in children aged less than 15 years appears to have high sensitivity (greater than 99%) across all time windows following the final transmission event (table 1). However, it had low specificity in our models: in 96% of scenarios, it was achieved before the final transmission event had actually occurred; with the majority of scenarios first passing phase 1 more than 10 years before the final transmission event (median: 11 years).

**Table 1.** Sensitivity of milestones. Five-, 10-, 15- and 20-year sensitivity for halted transmission and zero incidence. Rather than sensitivity, ‘less than 0 year’ represents the percentage of scenarios where a phase was misleadingly achieved prior to the final transmission event.

phase (milestone for progressing out of phase)		sensitivity			
	<0 year	5 year	10 year	15 year	20 year
phase 1 (5 years no child cases)	*96*.*3%*	99.2%	99.8%	100%	100%
phase 2 (3 years no cases)	*46*.*2%*	72.0%	91.1%	99.1%	100%
phase 3 (10 years only sporadic cases)	*1*.*0%*	12.8%^a^	41.4%^a^	71.1%	91.1%

^a^For scenarios where the milestone for passing out of phase 3 is achieved less than 15 years post the final transmission event, the last transmission must occur after the start of the 5-year window for achieving the milestone for passing out of phase 2 and is therefore falsely classified as a sporadic or non-autochthonous case.

The milestone for passing out of phase 1, used in isolation, could therefore result in a number of ongoing transmission events being falsely classified as sporadic or non-autochthonous cases. Despite this, reversal due to detecting more than two child cases in the same year was very rare and occurred in only 0.5% of simulations that achieved the milestone for passing out of that phase despite ongoing transmission. The probability of reversal after correctly achieving the milestone for passing out of that phase was also 0.5%, indicating that passing out of phase 1 and any subsequent reversal may not be a very helpful marker without the additional milestones.

Subsequently, achieving the milestone for passing out of phase 2 by recording 3 years of no autochthonous cases in adults or children, either after or alongside 5 years of no cases in children, also appeared to be sensitive, with a 72% chance of achieving the milestone for passing out of phase 2 within 5 years of transmission interruption. Given a 10-year or 15-year window, sensitivity increased to 91% and 99%, respectively.

However, in 46% of scenarios, the milestone for passing out of phase 2 was achieved before the final transmission event. Reversal in this case was due to detecting more than two cases in adults or children in the same year and was slightly more common, but still occurred in only 11.3% of simulations that achieved the milestone while transmission was ongoing. In comparison, 4.7% of scenarios that correctly achieved the milestone experienced reversal.

Passing out of phase 3, which requires 10 years of only sporadic autochthonous cases after previously passing out of phase 2, had low sensitivity on short time frames after transmission interruption, which reflects the fact that a minimum of 15 years must pass before achieving this milestone. The 15-year sensitivity is comparable to the 5-year sensitivity of passing out of phase 2 (71% compared to 72%) and is feasibly the earliest this milestone could be achieved without misleadingly achieving the milestone for passing out of phase 1 and/or phase 2 prior to the final transmission event. Sensitivity increases to 91% at 20 years post the final transmission event.

By contrast, there is a very low chance (around 1%) of passing out of phase 3 prior to interrupting transmission and relatively low chance of prematurely passing out of phase 3 at the 5-year and 10-year sensitivity marks (13% and 41%, respectively). There is also a very low risk of reversal, with less than 0.5% chance of reversal if the milestone has been correctly passed. However, if the milestone is achieved falsely, prior to transmission interruption, there is only a 4% chance of reversal to either phase 1 or phase 2.

### (b) Scenario 2. Low-level persistence

For simulations of a scenario representing low-level persistence (mean annual incidence of two new infections per year within an EU), the probability of passing out of phase 1 is high despite ongoing transmission. The 5-year and 10-year specificity estimates are 20% and 12%, respectively, meaning that there is an 80% chance of passing out of phase 1 in any given 5-year period while transmission was ongoing and an 88% chance across any 10-year period (table 2). When considering longer time frames, this specificity drops even further, to 4.5% across a 20-year period. There is also a relatively low chance of reversal, with only 3.5% of scenarios reversing across a 10-year period.

**Table 2.** Specificity of phases 10-, 15- and 20-year specificity of milestones for a low-level persistence scenario with a mean annual incidence of two new infections per year.

phase (milestone for progressing out of phase)	specificity			
	5-year	10-year	15-year	20-year
phase 1 (5 years no child cases)	20.3%	12.2%	6.7%	4.5%
phase 2 (3 years no cases)	95.1%	91.7%	87.7%	84.2%
phase 3 (10 years only sporadic cases)	99.9%	99.7%	99.5%	98.9%

The milestones for passing out of phase 2 and phase 3 have much higher specificity, with a 10-year specificity of 92% for the milestone for passing out of phase 2 and a 20-year specificity of 99% for the milestone for passing out of phase 3. There is also a much higher chance of reversal if these milestones are achieved while transmission is ongoing, with 17% of scenarios seeing a reversal within only 2 years of passing out of either phase. In the longer term, 54–55% of scenarios will reverse within 5 years and 82–85% will reverse within 10 years.

**Figure 4.**
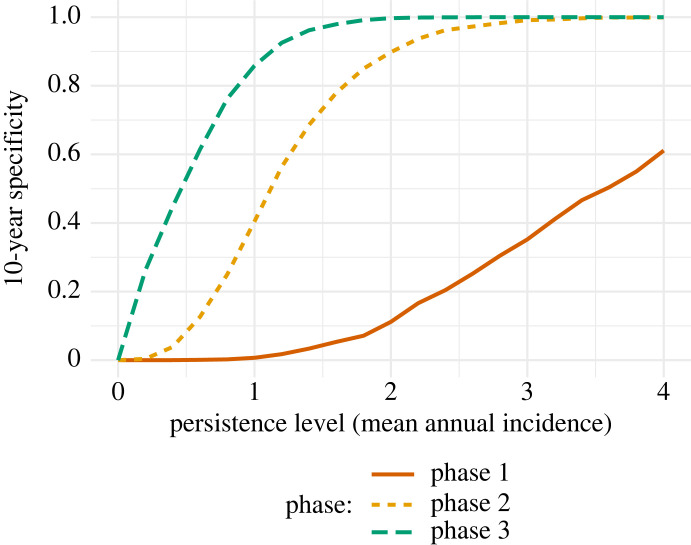
Ten-year specificity. The 10-year specificity of the milestones for the three phases of classifying elimination in the presence of persistent transmission (mean annual incidence).

The specificity—and reversal rate—of each milestone is dependent on the assumed level of incidence in any scenario of low-level persistence (figure 4). The 10-year specificity is poor (0–50%) for the milestone for passing out of phase 1 for the range of mean incidence considered (up to 4 cases per year), but better (greater than 75%) for the milestones for passing out of phase 2 and phase 3 for all but very low mean incidence. It is also important to remember that any scenario where specificity is lower, due to lower mean incidence, will also have a lower reversal rate.

A sensitivity analysis was conducted around the mean values of the incubation period and detection delay distributions, which can be seen in the electronic supplementary material.

## 4. Discussion

We have investigated the utility of a three-phase approach to classifying elimination of leprosy transmission, based on the WHO leprosy elimination monitoring tool, under two different epidemiological scenarios. The long incubation and detection delays associated with leprosy require case detection and treatment to be ongoing for 10 or more years following cessation of transmission, and an extended period of observation to verify non-endemic status. We have assessed both the effectiveness of the specified milestones of classifying elimination, as well as the timeliness of when these classifications occur.

It is important to note that this analysis is limited by our current knowledge of the incubation period of leprosy, informed by an importation study in American veteran soldiers, and the highly variable detection delays, which a recent analysis suggests are even more variable than previously thought [31]. In particular, previous analysis suggests that these delays are variable over time and across settings, with even longer delays likely recently due to reduced access to health services during the COVID-19 pandemic [11,12]. We have performed extensive sensitivity analysis, but these models are limited by the available data.

Our analysis is also based on simulating incidence across an EU, whereas, depending on the size of the EU, there may be multiple sub-epidemics within an EU, with some further from or closer to elimination. This further highlights the need for careful epidemiological monitoring, understanding and investigation as outlined in the technical guidelines.

In a scenario in which transmission was interrupted, we found that, while there was a 96% chance of achieving the milestone for passing out of phase 1 before transmission reached zero, there was only a 1% chance of passing out of all of phases 1–3 while transmission was ongoing. Additionally, we saw a 71% chance of achieving classification of elimination within 15 years of the final transmission event. As 15 years is the minimum length of time for passing out of all three phases, this represents reasonable sensitivity for detecting a halt in transmission. After another 5 years, 20 years after the final transmission event, this increases to a 91% chance. It is important to note that this is just one potential example of declining transmission and the rate of decline will have implications for the sensitivity estimates, but our results demonstrate that passing out of phase 1 is unlikely to be a strong indicator of interrupted transmission, even if passing out of all phases is a reasonable indicator of non-endemic status.

When considering scenarios representing persistent transmission, the specificity of the three classification milestones depended on the level of transmission and the period of time considered, with lower transmission scenarios and longer time periods giving a low specificity due to a higher chance of achieving the milestone for each phase with transmission ongoing. Although the milestone for passing out of phase 1 has low specificity (less than 35% across a 10-year period) in the persistence scenario demonstrated in table 2 (mean of two new infections per year at an EU level), the milestone for passing out of phase 2 demonstrates a much higher specificity, with less than 15% of scenarios passing out of phases 1–2 across a 20-year period while transmission was ongoing. In addition, the milestone for passing out of phase 3 is highly specific (greater than 99%) across the same period. However, if mean annual incidence is below two infections (per EU per year), these specificities may be lower.

Overall, our analysis suggests that the criterion for passing out of phase 1 (5 years of zero autochthonous cases in children) is unlikely to be a strong indicator of interruption of transmission, with a very high chance of false achievement and a low reversal rate, despite high sensitivity. This is due to the requirement of more than two cases in children in 1 year for reversal, which is unlikely at such low transmission levels. However, as child cases are a good indicator of more recent transmission, this is a useful criterion when used in combination with the other phases. Increasing the age from under 15 to under 18 would increase the size of this subset of the population and therefore potentially improve the specificity of this milestone but may have other biological implications.

The milestones for passing out of phases 2 and 3 are much better indicators of elimination, with much higher specificity (greater than 85% across a 20-year period at all but the lowest incidence levels), representing a lower chance of passing out of phases before, or in the absence of, elimination, plus a higher chance of reversal within a sensible timeframe (5–10 years) if this does occur. The milestone of passing out of phase 2 also has good sensitivity, with a 91% chance of achievement within 10 years post the final transmission event, even when allowing it to coincide with the milestone for passing out of phase 1, making it a potentially timely and effective milestone on the road to classifying elimination of leprosy transmission.

Passing out of phase 3 would require an additional 10 years, substantially extending the time frame of classification, but is a very good indicator of non-endemic status when used in combination with having already achieved the milestones for passing out of phases 1 and 2. It is highly specific in our model, with less than a 1% chance of achievement while transmission was ongoing over a 20-year period of low-level persistence and is reasonably sensitive across the minimum achievement time period of 15 years.

Together, the three phases represent a staged, effective and relatively timely indicator of transmission interruption. The minimum 15-year period (18 years if phases 1 and 2 do not overlap) is sufficiently long to cover the majority of incubation and detection delays, with the allowance of sporadic cases in and beyond the final 10-year duration of phase 3 ensuring that cases detected at the tail of these distributions do not undermine programme achievements. This is reasonably consistent with previous estimates that for 95% of individuals onset will occur within 17.8 years and detection will occur within 23.6 years of infection [12].

We have focused on one specific example of milestones in the 2023 leprosy elimination technical guidelines, allowing us to present detailed estimates of specificity and sensitivity for this example, but other milestones could also be used. Using cases in children as a proxy for more recent transmission provides a first step for programmes looking to demonstrate to stakeholders that they are making progress and on the right track. This can then be followed by more stringent requirements, such as are in the guidelines for passing out of phases 2 and 3.

There are several requirements we consider important for any elimination classification process. First, there needs to be consideration of how programmes can clearly demonstrate ongoing progression towards the target, as is outlined in the new guidelines. Second, the time frames involved should be sufficiently long (minimum 15 years) to capture the majority of delays between transmission and case detection, as well as longer term allowance for sporadic or non-autochthonous cases, to avoid the chance of historic infections undermining programme achievements. Third, there should be a clear understanding of what each milestone represents in terms of the likelihood that non-endemic status has been achieved, to aid public health understanding and policy decisions around ongoing detection efforts.

Our analysis only considers two independent scenarios: exponential decline to zero, and low-level persistence (at a defined mean annual incidence). It is possible that other scenarios, such as a slow increase in transmission or fluctuating levels of incidence, could occur. In the case of a slow increase in transmission, this might not be detected for a number of years, but should substantially decrease the probability of achieving the milestones for passing out of phases 2 and 3 while transmission is ongoing and increase the chances of reversal as time goes on. For larger fluctuations in incidence than those considered in this study, we might expect to see more misleading achievement of milestones, even of phase 2 or 3 if these fluctuations are slow, but we would also expect to see a much higher rate of reversal, which should alert the programme that there is cause to be concerned.

Due to the low number of cases in low-incidence settings, we were unable to fit a full transmission model. However, looking at trends from parts of countries close to elimination, where there has been low-level incidence of detection (less than 10 cases per year) over a 20-year period [22], the main low-level persistence scenario (a mean incidence of two infections per year) appears to best describe the level of fluctuations seen in the data (see electronic supplementary material, figure S1). As a consequence, we have focused on this scenario in tables 1 and 2.

We also conducted a sensitivity analysis around the incubation period and detection delay distributions to consider the impact of likely different distributions in different settings [31], and found that uncertainty in incubation period had a larger potential to affect model output than uncertainty in detection delay, probably due to the longer relative duration of the incubation period. However, our results remained qualitatively similar even when considering a range of mean incubation period between 3.9 and 11.7 years, and mean detection delays ranging from 1.1 to 3.4 years. Overall, longer delays did lead to higher risk of achieving milestones while transmission was ongoing, but the risk of passing out of phases 2 and 3 despite ongoing transmission remained relatively low across all scenarios (full details in the electronic supplementary material).

There are still substantial challenges associated with the timely detection of leprosy cases and transmission. The next few years will be vital in terms of gathering data and evidence for how elimination of leprosy presents from a programmatic perspective. However, we believe we have shown here that, if implemented with a balanced and comprehensive understanding of what each one represents, the combined phases and milestones outlined in the WHO technical guidance are likely to effectively classify elimination of leprosy transmission.

## Data Availability

All code used in the study is publicly available and released from the Zenodo repository: https://zenodo.org/record/8238792. The data are provided in electronic supplementary material [32].
